# Amygdala Changes in Chronic Insomnia and Their Association with Sleep and Anxiety Symptoms: Insight from Shape Analysis

**DOI:** 10.1155/2019/8549237

**Published:** 2019-12-01

**Authors:** Liang Gong, Tianzhi Liao, Duan Liu, Qiuhua Luo, Ronghua Xu, Qun Huang, Bei Zhang, Fen Feng, Chuantao Zhang

**Affiliations:** ^1^Department of Neurology, Chengdu Second People's Hospital, Chengdu, Sichuan 610017, China; ^2^Hospital of Chengdu University of Traditional Chinese Medicine, Chengdu, Sichuan 610072, China; ^3^Department of Respiratory Medicine, Hospital of Chengdu University of Traditional Chinese Medicine, Chengdu, Sichuan 610072, China

## Abstract

Affective disorders, such as anxiety and depression, are common comorbidities associated with chronic insomnia disorder (CID). However, the underlying neural mechanisms of these comorbidities are still not clear. The present study is aimed at investigating structural changes in the amygdala of CID patients using surface-based shape analysis. A total of 65 medication-naive patients with CID and 55 healthy controls (HCs) matched for age, sex, and years of education were enrolled in this study and were subjected to structural magnetic resonance imaging (MRI). The Oxford Centre for Functional MRI of the Brain (FMRIB) created an Integrated Registration and Segmentation Tool (FIRST) that was employed in this study to assess the volumetric and surface alterations in patients with CID. Shape correlations between the amygdala and clinical features were also analyzed. Atrophic changes in the amygdala were observed at the local level, not for the entire amygdala volume. The left atrophic changes in the amygdala were in the superficial and basolateral nuclei while right atrophic changes were in the basolateral nuclei in CID patients. Insomnia severity was associated with the centromedial right amygdala while anxiety was linked with the basolateral nuclei. These findings indicate localized amygdala atrophy in CID. Separate amygdala regions are associated with insomnia and anxiety in CID. This evidence helps elucidate the neural mechanisms underlying the bidirectional relationship between insomnia and anxiety.

## 1. Introduction

Chronic insomnia disorder (CID) affects an estimated 10% of the population and is associated with an impaired quality of life and the presence of mental health disorders, especially affective disorders and suicidal behavior [1–3]. Anxiety and depression are affective symptoms that are frequently experienced in patients with CID. However, for many patients suffering from depression and anxiety, insomnia is a pervasive problem. Several longitudinal epidemiological studies have indicated that insomnia is bidirectionally related to anxiety and depression [4–6]. Despite insomnia being widely associated with affective disorders, the neuropathology of comorbid anxiety and depression in CID is still poorly understood. In particular, many of the etiological theories have suggested that heightened emotional reactivity may play a key role in the pathophysiology of insomnia disorder and its associated affective symptoms [6, 7].

The amygdala is located in the medial temporal lobe and plays a key role in emotional processing [8, 9]. Serving as a hub of emotional circuits, the amygdala participates in strong bidirectional interactions with the prefrontal and anterior cingulate cortex and is involved in emotional regulation; it also receives inputs from subcortical neurons including gamma-aminobutyric acid (GABA)ergic, dopaminergic, serotonergic, and noradrenergic neurons associated with the arousal system [10, 11]. Amygdala dysfunction has been implicated in the neuropathology of a majority of emotional disorders such as schizophrenia, social anxiety disorder, major depressive disorder, and obsessive-compulsive disorder [12–14], as well as insomnia [7, 15]. Recently, several neuroimaging studies have revealed abnormal amygdala function in chronic insomnia. For instance, Baglioni et al. demonstrated that patients with insomnia show amygdala hyperactivity in response to sleep-related stimuli [7, 16]. Using a resting state functional magnetic resonance imaging (fMRI) method, Huang et al. found that the amygdala-based intrinsic emotional network is abnormal in patients with primary insomnia and the functional connectivity between the amygdala and premotor neurons is positively correlated with insomnia severity [15]. With regard to structural neuroimaging, previous studies have shown a decreased brain volume in the medial frontal and middle temporal gyrus, middle cingulate cortex, and hippocampus using voxel-based morphometry (VBM) [17–19]; however, these studies do not show any significant alterations in the amygdala. More recently, Koo et al. used a FreeSurfer-based analysis and reported atrophy in subcortical structures including the hippocampus, amygdala, basal ganglia, and thalamus in patients with chronic insomnia; nonetheless, the study did not report the details of localized shape alterations or the association between shape alteration and emotional symptoms [20]. To our knowledge, no study has investigated amygdala shape alteration and its association with emotional features in patients with CID. Accordingly, elucidating the relationship between the altered amygdala structure and affective symptoms in patients with CID requires further investigation.

The Oxford Centre for Functional MRI of the Brain (FMRIB) developed an Integrated Registration and Segmentation Tool (FIRST); it is a new automated structural MRI analysis tool available in the FMRIB software library (FSL) for subcortical nuclei. FIRST can achieve an individual level segmentation to the outer surface of substructure nuclei [21]. FIRST segmentation of the amygdala is comparable with expert manual segmentation and offers advantages in small subcortical structures in a scan-rescan [22, 23]. Furthermore, FIRST evaluates structural volume alterations and can be used for shape analysis as it can examine local changes in the subcortical nuclei. Thus, it is a useful tool for amygdala morphometric analysis in neural mechanism research, especially in neuropsychiatric diseases [24, 25].

In the present study, FIRST was used along with shape analysis to investigate potential structural alterations of the amygdala in patients with CID compared to healthy controls (HCs) with adequate, quality sleep. Also, a surface-based vertex-wise correlation analysis was used to detect local associations between amygdala morphology and insomnia severity as well as affective symptoms (anxiety and depression) in CID patients. We hypothesized that amygdala atrophy would be observed locally in the CID group; moreover, the amygdala shape, not its volume, would be associated with insomnia and affective symptoms in patients with CID.

## 2. Materials and Methods

### 2.1. Participants

A total of 65 medication-naive patients with CID and 55 HCs matched for age, sex, and years of education were enrolled in the present study. In the enrollment period, all participants completed a self-rated medical history questionnaire and underwent a structured clinical interview by 2 senior trained neurologists (LG and DL) according to the 3^rd^ version of International Classification of Sleep Disorders (ICSD) and the Diagnostic and Statistical Manual of Mental Disorders, Fifth Edition (DSM-V). Patients with CID met the following inclusion criteria: [1] diagnostic criteria for CID according to the 3^rd^ version of the ICSD [26]; [2] at least 3 months of difficulty falling asleep, maintaining sleep, or early wakening; [3] no hypnotic or antidepressant medication used in the 2 weeks before the neuropsychological and MRI assessments; and [4] aged 18–55 years with an age of insomnia onset under 50 years. The exclusion criteria for the CID group were as follows: [1] history of another neuropsychiatric disorder, such as major depressive disorder or general anxiety; [2] other sleep disorders, such as sleep-related breathing disorders (sleep apnea syndrome), central disorders of hypersomnolence, circadian rhythm sleep-wake disorders, sleep-related movement disorders, parasomnia, or hypersomnia; [3] abuse of substances such as caffeine and alcohol [27]; [4] contraindications to MRI; and [5] a brain lesion detected during T2-weighted image (T2WI) MRI. HCs were required to meet the following criteria: [1] quality sleep, positive mood, and normal cognitive function; [2] no history of neurological or psychiatric disease, seizures, head injury, stroke, or transient ischemic attack; [3] no caffeine, drug, or alcohol abuse; and [4] no brain lesions detected by T2WI MRI. The study was approved by the Institutional Review Board of the Hospital of Chengdu University of Traditional Chinese Medicine. All participants signed the informed consent form.

### 2.2. Assessments

The Pittsburgh Sleep Quality Index (PSQI) was employed to evaluate insomnia severity in the CID group [28, 29]. The self-rating depression scale (SDS) was used to assess depression and the self-rating anxiety scale (SAS) was used to measure anxiety.

### 2.3. Image Acquisition

MRI data were acquired between 4 p.m. and 6 p.m. for all participants. Imaging was performed using a 3.0-Tesla MRI scanner (GE Healthcare Discovery MR750, Milwaukee, WI, USA) equipped with an 8-channel head coil. Structural images were acquired using a high-resolution, spoiled gradient-recalled echo with the following parameters: repetition time = 2900 ms, echo time = 2.48 ms, flip angle = 7°, acquisition matrix = 256 × 256, field of view = 256 × 256 mm^2^, thickness = 1.0 mm (with no gap), number of slices = 188, and voxel size = 1 × 1 × 1 mm^3^.

### 2.4. Image Preprocessing

MRI data analyses were performed using the tools from the FSL (version 5.0.9, https://fsl.fmrib.ox.ac.uk/fsl; FMRIB Software Library, Oxford University, Oxford, UK) [30]. The default parameters in the FSL were used. Before the subcortical region segmentation, the total intracranial volume (TIV), white matter volume (WMV), and gray matter volume (GMV) for each participant were estimated using SIENAX (part of the FSL, https://fsl.fmrib.ox.ac.uk/fsl/fslwiki/SIENA). The SIENAX method includes 4 steps: [1] brain and skull images are extracted from the input image; [2] the brain images are affine-registered to standard Montreal Neurological Institute (MNI) 152 space; [3] tissue-type segmentation with partial volume estimation is performed to acquire TIV, WMV, and GMV; and [4] brain volumes are standardized to a “normalized” skull size [31].

Then, the amygdala and other subcortical volume and shape information were preprocessed by FIRST (https://fsl.fmrib.ox.ac.uk/fsl/fslwiki/FIRST, part of the FSL, version 5.0.9) [21], an automated tool that can segment the subcortical nuclei and has been used to study several neuropsychiatric disorders [24, 25]. This approach is based on Bayesian statistical models; the shape and appearance of subcortical structures are constructed from 336 manually labeled brain images provided by the Center for Morphometric Analysis, Massachusetts General Hospital, Boston. FIRST processing consists of 4 steps: [1] raw individual brain images are registered to MNI152 space by 12 degrees of freedom and accurately registered to a subcortical mask; [2] subcortical structures are established by deformable meshes consisting of vertices and edges; [3] subcortical structures are automatically segmented based on a Bayesian framework; [4] then both surface mesh and volumetric outputs are generated; [5] the quality of segmentation for each person was checked manually (2 patients with CID were excluded). Afterwards, FIRST outcome files were used for volume and surface-based vertex analyses.

### 2.5. Surface-Based Shape Analysis

A new vertex-wise analysis algorithm was employed to investigate localized shape differences in the bilateral amygdala adjusting for age and sex (first_utils and *“Randomise” algorithm*, FSL 5.0.9). General linear models and permutation testing utilized the “Randomise” FSL module [32]. This approach calculates the group differences on a per-vertex basis. The threshold-free cluster enhancement (TFCE), a new method for finding significant “clusters” in a statistical image without defining clusters in a binary manner, was used to identify vertex clusters with significant shape deformations in CID with a family-wise error (FWE) rate included for multiple comparison correction [33]; the FWE-corrected *p* values were set as the default (*p* < 0.05). As the traditional surface-based vertex analysis comprises the vectors in each significant vertex, it was used to display the direction of group differences. In groups demonstrating TIV differences, an additional analysis was conducted to verify whether the results remained significant after including total intracranial volume as an additional covariate.

Furthermore, a vertex-wise correlation analysis was employed to detect potential associations between amygdala morphology and clinical features (duration of disease, PSQI, SAS, and SDS scores) in the CID group, controlled for age and sex. A significant clinical feature and amygdala shape correlation assessment was also conducted by using the “Randomise” algorithm with TFCE and FWE correction. Additionally, as the hippocampus is most often reported to have an abnormal subcortical structure in CID [18, 34, 35], group differences in bilateral hippocampal shape were calculated using the same method.

### 2.6. Statistical Analysis

A two-sample *t*-test was conducted to compare various demographic data between the 2 groups, while the chi-square test was used to compare data between sexes. An analysis of covariance was used to estimate group differences in the volume of the entire brain (WMV, GMV, and TIV), bilateral amygdala volume, and bilateral hippocampal volume, with age and sex as covariates (SPSS 20, Inc., Chicago, IL, USA). A Pearson correlation was employed to examine the relationship between bilateral amygdala and hippocampal volumes; disease duration; and the PSQI, SAS, and SDS scores in the CID group. Statistical significance was set at *p* < 0.05, adjusted for multiple comparisons using a Bonferroni correction (*p* < 0.00625 = 0.05/8).

## 3. Results

### 3.1. Demographic Information and Clinical Features

As shown in Table 1, no significant group differences were found in age, sex, or years of education (*p* > 0.05). In the CID group, disease duration was not significantly correlated with PSQI, SDS, or SAS scores (all *p* > 0.05), and PSQI results were not significantly correlated with those of the SDS (*r* = 0.09, *p* = 0.47), but were positively associated with SAS scores (*r* = 0.35, *p* = 0.004). Moreover, SDS scores were positively correlated with SAS scores in the CID group (*r* = 0.36, *p* = 0.003).

### 3.2. Brain Volumes

The TIV and WMV showed no significant difference between the 2 groups. The GMV in the CID group was smaller than that in the HC group (*p* = 0.02). There was no significant difference in bilateral amygdala or hippocampal volumes between CID and HC participants (Table 1). The partial correlation analyses revealed that the right amygdala volume was negatively correlated with PSQI scores in the CID group (*r* = −0.287, *p* = 0.021); however, the correlation was not significant after multiple comparison correction.

### 3.3. Shape Analysis: Group Comparisons

**Figure 1:**
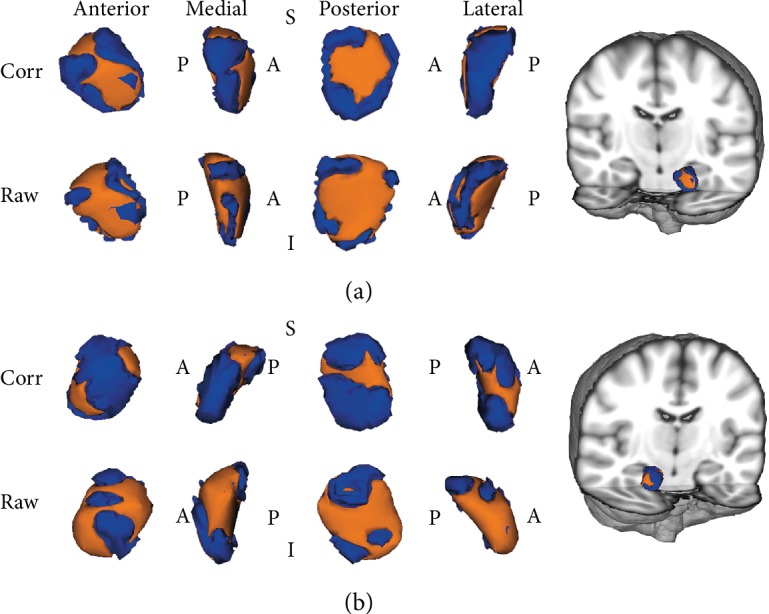
The localized shape differences between HC and CID groups using vertex-wise surface analyses of the amygdala are shown. The regions in orange indicate the areas shown to be smaller in patients with CID than in those in HC group. (a) The left amygdala group differences are in the superficial and basolateral nuclei of the amygdala. (b) The right amygdala group differences are in the basolateral nuclei of the amygdala. Abbreviations: CID: chronic insomnia disorder; HC: healthy control; Corr: TFCE corrected; Raw: raw results; TFCE: threshold-free cluster enhancement; A: anterior; P: posterior; S: superior; I: inferior.

**Figure 2:**
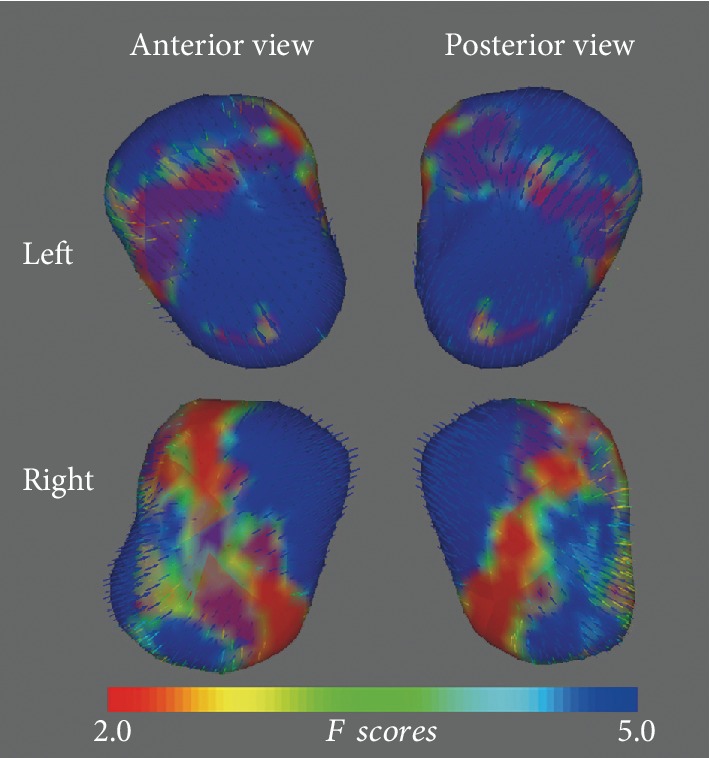
Vector graphs of the amygdala according to a traditional surface-based vertex analysis displayed by three-dimensional mesh are shown. The color bar indicates the statistic values; as the color increases from red to blue, a lower to higher statistical significance is indicated. The small arrows shown on the surface indicate the direction of change. Inward arrows indicate that the diseased amygdala is smaller/thinner in that location than it is in the healthy control group. Abbreviations: Left: left amygdala; Right: right amygdala.

As shown in Figure 1, vertex analysis revealed that the superficial (SF) and basolateral (BL) nuclei of the left amygdala and the BL nuclei of the right amygdala had significant group differences in CID patients compared to HCs (TFCE corrected). The traditional surface-based vertex analysis showed an inward displacement in these significantly different regions of the amygdala (Figure 2), while the shape analysis findings indicated localized amygdala atrophy in the CID group compared to the HC group. No significant areas of hypertrophy were observed. Furthermore, no significant group differences were found via bilateral hippocampus shape analysis. The additional analysis showed the same results whether age, sex, and TIV were used as covariates in the group comparisons.

### 3.4. Shape Analysis Correlations

**Figure 3:**
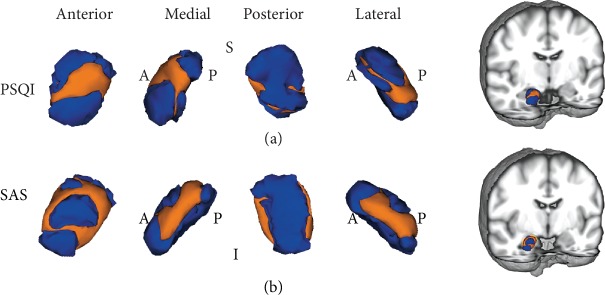
The localized shape association of the right amygdala with PSQI and SAS scores in the CID group is shown. The regions in orange indicate the area associated with the PSQI score (a) and SAS score (b) in the CID group. Abbreviations: PSQI: Pittsburgh Sleep Quality Index; SAS: self-rating anxiety scale; CID: chronic insomnia disorder; A: anterior; P: posterior; S: superior; I: inferior.

**Figure 4:**
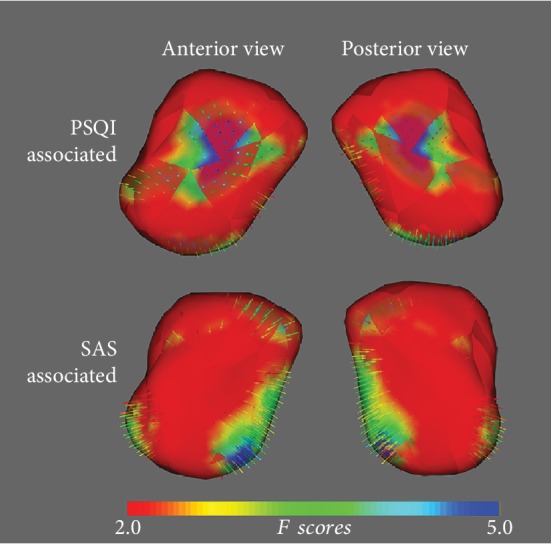
Vector graphs of the right amygdala associated PSQI and SAS scores according to a traditional surface-based vertex correlation analysis displayed by three-dimensional mesh are shown. The color bar indicates the statistical values; as the color changes from red to blue, statistical significance changes from low to high. The small arrows on the surface indicate the direction of association. Inward arrows indicate the PSQI/SAS score was negatively correlated with the regional vertex of the right amygdala. Abbreviations: PSQI: Pittsburgh Sleep Quality Index; SAS: self-rating anxiety scale.

Figure 3 illustrates the correlation of localized amygdala shape with PSQI and SAS scores. As demonstrated in Figure 4, the inward correlation indicates a negative association of the right amygdala shape with PSQI and SAS scores in the CID group. Interestingly, the PSQI-associated region was mainly in the centromedial (CM) amygdala, while the SAS-associated region was in the BL amygdala. These results suggest that different neural mechanisms may be responsible for insomnia and anxiety in CID. There were no correlations between the left amygdala shape and clinical features in the CID group. Disease duration and SDS scores showed no significant association with bilateral amygdala shape in the CID group.

## 4. Discussion

The present study investigated the potential association of amygdala morphology alterations with insomnia and emotional symptoms in patients with CID using vertex-based shape analysis. The key results are as follows: [1] atrophic changes in the amygdala are local, not encompassing the whole amygdala, in CID; [2] the left atrophic amygdala was in the SF and BL nuclei while the right atrophic amygdala was in the BL nuclei in patients with CID; [3] both insomnia and anxiety were associated with the right amygdala shape but were independent of the nuclei. Taken together, these findings indicate localized amygdala atrophy in patients with CID. Moreover, the subregional, specialized association of the amygdala with insomnia and anxiety will help elucidate the neural mechanisms underlying the bidirectional relationship between these 2 CID characteristics.

Localized shape analysis of the subcortical nuclei is a relatively new approach to detect structural alterations in neuropsychiatry diseases; accordingly, it has rarely been implemented in CID research. The present volumetric analysis did not show significant group differences between CID and HC groups; however, regional bilateral amygdala atrophy was detected by shape analysis. The present findings are not fully consistent with the previous structural findings obtained via the VBM approach for the GMV or the overall amygdala volume [17, 18]. Methodologically, the VBM approach is considered a sensitive method to explore cortical structure changes; however, due to the poor and variable intensity contrast in subcortical structures, it has a limited ability to precisely localize atypical brain region alterations [36]. The present results indicate that the overall amygdala volume estimate may not capture structural alterations in patients with CID. Thus, we propose that a shape-based morphology analysis could be a useful tool to detect early subcortical atrophy in CID.

Histologically, the amygdala is composed of 3 main nuclear complexes: the BL, CM, and SF nuclei [37]. Recently, these 3 clusters have been investigated in the human brain by connectivity-based parcellation using diffusion and resting-state functional MRI data [38]. The amygdala is anatomically connected with and functionally modulates the brainstem centers involved in arousal and sleep regulation [11, 39, 40]. Recent studies have established amygdala dysfunction in patients with insomnia [7, 15, 41]. However, due to the low structural resolution of the subcortical nucleus, these functional neuroimaging studies on insomnia did not report detailed information about alterations in amygdala subregions. The 3T structural MRI-based shape analysis, used in the present study, provides more information about the amygdala alterations in insomnia. Consistent with our hypothesis, the findings show that amygdala atrophy was present in the left SF and BL nuclei as well as the right BL nuclei in patients with CID. The BL amygdala nuclei are important for environmental information processing and self-relevant cognition integration, while SF nuclei are highly tuned to social information processing [38]. We speculate that the observed amygdala atrophy in SF and BL nuclei might reflect abnormal environmental information processing, self-relevant cognition, and social information in CID patients.

Patients with CID frequently experience intrusive thoughts during the sleep-onset period. According to the 2 component theory of intrusive thoughts, “rumination” is associated with a negative mood, while “worry” is linked to an anxious mood and involves catastrophizing about stressful events [42, 43]. In the present study, a single dissociation of affective states was associated with amygdala subregion nuclei in patients with CID. Namely, anxiety, not depression, was associated with right amygdala shape alterations in the insomnia group. This result supports the pathway of emotional valence in the cognitive activity of insomnia and indicates that the amygdala has a role as a mediator in the association between anxiety and insomnia [6].

The CM amygdala nuclei have large numbers of GABAergic neurons and have direct projections to the hypothalamus, brainstem, and ascending cholinergic and monoaminergic systems [11, 44]. Functionally, the CM nuclei play a major role in autonomic arousal regulation [38]. Thus, the association of the PSQI score with the right CM amygdala nuclei in the present study supports the notion that the CM nuclei are involved in sleep regulation. Additionally, the BL amygdala nucleus extensively projects to the prefrontal cortex, medial temporal lobe, and striatum; it receives and integrates sensory information and assigns an affective value to the stimuli [45, 46]. The present finding of anxiety being associated with atrophy in the BL nuclei indicates abnormal integration and assignment function in patients with CID. Importantly, this study demonstrated a dissociation between the right amygdala subregion and the severity of insomnia and anxiety symptoms in CID. Therefore, these findings signify that although a clinical relationship between insomnia and anxiety severity is observed in CID, there may be different underlying neural mechanisms for the 2 comorbid conditions.

The present study has several limitations. First, the results of this cross-sectional design study cannot be interpreted as a causal relationship between amygdala shape and insomnia symptoms in patients with CID. Second, CID is a heterogeneous clinical disorder that includes different pathophysiology subtypes in terms of cognitive performance, emotional deficits, personality traits, childhood trauma, life events, and family history [47]; therefore, it would be difficult to find consistent brain alterations in different neuroimaging study modalities in insomnia [48, 49]. Future studies should enroll a more homogenous participant population or involve a homogenous analysis. Finally, anxiety and depression states in the present study were evaluated based on self-rating scales. Further research should employ more comprehensive neuropsychological tests to detect amygdala atrophy associated with the cognitive and emotional performance in CID.

## 5. Conclusion

The current study is the first to localize alterations in the amygdala associated with insomnia severity and anxiety states in patients with CID using surface-based shape analysis. The amygdala was atrophied in the SF and BL subregions in CID patients. Furthermore, the novel relationship revealed between localized amygdala atrophy, insomnia, and anxiety will help elucidate the underlying brain mechanisms of the bidirectional relationship between these 2 CID characteristics.

## Figures and Tables

**Table 1:** Demographic, clinical characteristics, and brain volume data for the two groups.

Characteristic	CID (*n* = 65)	HCs (*n* = 55)	*T*/*χ*^2^ value	*p* value
Age (years)	38.46 ± 11.38	41.03 ± 13.77	1.11	0.27
Gender (female/male)	42/25	23/27	3.22	0.07^†^
Year of education	13.35 ± 3.88	12.76 ± 3.29	0.86	0.38
Duration (months)	58.78 ± 63.07	—	—	—
PSQI score	13.92 ± 1.95	—	—	—
SDS score	53.50 ± 8.11	—	—	—
SAS score	53.55 ± 5.31	—	—	—
Gray matter volume	792.12 ± 95.96	827.01 ± 50.32	2.42	0.02
White matter volume	740.24 ± 83.36	753.78 ± 41.57	1.09	0.27
Total intracranial volume	1562.23 ± 172.77	1580.79 ± 83.35	0.79	0.35
Left amygdala volume	1.17 ± 0.26	1.12 ± 0.32	1.25	0.21
Right amygdala volume	1.18 ± 0.43	1.10 ± 0.37	0.66	0.38
Left hippocampus volume	3.56 ± 0.74	3.64 ± 0.76	0.95	0.24
Right hippocampus volume	3.60 ± 0.73	3.77 ± 0.54	1.44	0.15

Notes: ^†^the *p* value was obtained by a chi-square test; other *p* values were obtained by a two-sample *t*-test. Abbreviations: CID: chronic insomnia disorder; HC: healthy control; PSQI: Pittsburgh Sleep Quality Index; SDS: self-rating depression scale; SAS: self-rating anxiety scale.

## Data Availability

The data used to support the findings of this study are available from the corresponding author upon request.
